# Translation and validation of the Korean version of the clinical frailty scale in older patients

**DOI:** 10.1186/s12877-021-02008-0

**Published:** 2021-01-13

**Authors:** Ryoung-Eun Ko, Seong Mi Moon, Danbee Kang, Juhee Cho, Chi Ryang Chung, Yunhwan Lee, Yun Soo Hong, So Hee Lee, Jung Hee Lee, Gee Young Suh

**Affiliations:** 1 Department of Critical Care Medicine, Samsung Medical Center, Sungkyunkwan University School of Medicine, 81 Irwon-ro, Gangnam-gu, Seoul, 06351 Republic of Korea; 2 Division of Pulmonary and Critical Care Medicine, Department of Medicine, Samsung Changwon Hospital, Sungkyunkwan University School of Medicine, Changwon, Republic of Korea; 3 Center for Clinical Epidemiology, Samsung Medical Center, Seoul, Republic of Korea; 4 Department of Clinical Research Design and Evaluation, SAIHST, Sungkyunkwan University, Seoul, Republic of Korea; 5 Department of Epidemiology, Johns Hopkins University Bloomberg School of Public Heath, Baltimore, MD USA; 6 Department of Preventive Medicine & Public Health, Ajou University School of Medicine, Suwon, Republic of Korea; 7 Institute on Aging, Ajou University Medical Center, Suwon, Republic of Korea; 8 Departments of Epidemiology and Medicine, and Welch Center for Prevention, Epidemiology, and Clinical Research, Johns Hopkins University Bloomberg School of Public Health, Baltimore, MD USA; 9 Outpatient Nursing Team, Samsung Medical Center, Seoul, Republic of Korea; 10 Medical Intensive Care Unit, Samsung Medical Center, Seoul, Republic of Korea; 11 Division of Pulmonary and Critical Care Medicine, Department of Medicine, Samsung Medical Center, Sungkyunkwan University School of Medicine, Seoul, Republic of Korea

**Keywords:** Clinical frailty scale, Frailty, Translation, Validation, Korean

## Abstract

**Background:**

Frailty is a multidimensional syndrome that leads to an increase in vulnerability. Previous studies have suggested that frailty is associated with poor health-related outcomes. For frailty screening, the Clinical Frailty Scale (CFS) is a simple tool that is widely used in various translated versions. We aimed to translate the CSF into Korean and evaluated its contents and concurrent validity.

**Methods:**

Translations and back-translations of the CFS were conducted independently. A multidisciplinary team decided the final CFS-K. Between August 2019 and April 2020, a total of 100 outpatient and inpatient participants aged ≥65 years were enrolled prospectively. The clinical characteristics were evaluated using the CFS-K. The CFS-K scores were compared with those of other frailty screening tools using Pearson’s correlation coefficient and Spearman’s rank correlation. The area under curve (AUC) for identifying the Eastern Cooperative Oncology Group Performance Status (ECOG PS) grade 3 or more was calculated for the CFS-K and other screening tools.

**Results:**

The mean age of the participants was 76.5 years (standard deviation [SD], 7.0), and 63 (63%) participants were male. The mean CFS-K was 4.8 (SD, 2.5). Low body mass index (*p =* 0.013) and low score on the Korean version of the Mini-Mental State Examination (*p <* 0.001) were significantly associated with high CFS-K scores, except for those assigned to scale 9 (terminally ill). The CFS-K showed a significant correlation with other frailty screening tools (R = 0.7742–0.9190; *p <* 0.01), except in the case of those assigned to scale 9 (terminally ill). In comparison with other scales, the CFS-K identified ECOG PS grade 3 or more with the best performance (AUC = 0.99). Patients assigned to scale 9 on the CFS-K (terminally ill) had similar frailty scores to those assigned to scale 4 (vulnerable) or 5 (mildly frail).

**Conclusions:**

In conclusion, the CFS-K is a valid scale for measuring frailty in older Korean patients. The CFS-K scores were significantly correlated with the scores of other scales. To evaluate the predictive and prognostic value of this scale, further larger-scale studies in various clinical settings are warranted.

## Background

Frailty is a multidimensional syndrome involving loss of reserves (energy, physical ability, cognition, and health) accompanied by an increase in vulnerability to increased dependency and/or mortality when exposed to a stressor [1, 2]. Frailty is either physical or psychological or a combination of both [2]. Physical frailty is characterized by diminished strength and endurance and reduced physiologic function [1] and associated with increased health-related outcomes in older populations, including hospitalization, nursing home admission, re-admission, and mortality [3–10]. Therefore, for physicians, frailty screening is useful for risk stratification, goal setting and advanced care planning, and frailty-targeted interventions [1, 11–13].

A recent consensus conference which was attended by the international societies and experts in the area of frailty recommended screening for frailty in all older persons and individuals with significant weight loss due to chronic disease [1]. They also suggested instruments for several screening tests such as the Fatigue, Resistance, Ambulation, Illness, and Loss of weight (FRAIL) questionnaire, Cardiovascular Health Study (CHS) frailty screening, and Clinical Frailty Scale (CFS) [1, 14]. Among them, the CFS is the most widely applied assessment tool [15]. The CFS is a simple, rapid screening test proposed by Rockwood and colleagues [2]. The CFS was based on the theoretical model of fitness, frailty, and function; it was developed as a grading tool with seven scales in 2005 [2] and revised in 2008 to include a total of nine scales. The CFS is composed of visual and written charts for frailty with nine graded pictures [2] and it takes less than 5 min to complete [14]. The CFS was developed to measure the frailty based on clinical judgement [2, 14], and studies have shown that CFS is useful to predict clinical outcomes in various clinical settings such as emergency department, intensive care units or postoperative [5, 16–19]. Because of its usefulness, the original English version of the CFS has been translated in different languages [12, 20, 21].

In Korea, The Eastern Cooperative Oncology Group Performance Status (ECOG PS) scale, the Korean version of FRAIL (K-FRAIL), Korean Cancer Study Group Geriatric Score (KG-7), and Korean Frailty Index are commonly used to assess frailty due to lack of appropriate measures [22–25]. However, these tools require more time for completion than the CFS, and most are limited to cancer patients. Herein, we aimed to validate the Korean version of the CSF (CSF-K). Specifically, we translated the CSF into Korean and evaluated its contents validity. In addition, we also evaluate specificity and sensitivity of the CSF-K and concurrent validity by comparing with other scales.

## Methods

### Participants

We prospectively enrolled 100 patients aged ≥65 years who visited an outpatient clinic or were admitted to the general ward or intensive care units of the Samsung Medical Center and Samsung Changwon Hospital between August 2019 and April 2020. The patients were eligible to participate if they or their guardians, who were closely involved in their care, gave informed consent to measure frailty. Patients diagnosed with dementia were excluded. The Institutional Review Board of the Samsung Medical Center (IRB No. 2019–02–028-004) and Samsung Changwon Hospital (IRB No. 2019–06-003) approved this study, and each participant provided informed written consent.

### Translation of clinical frailty scale to Korean

**Fig. 1:**
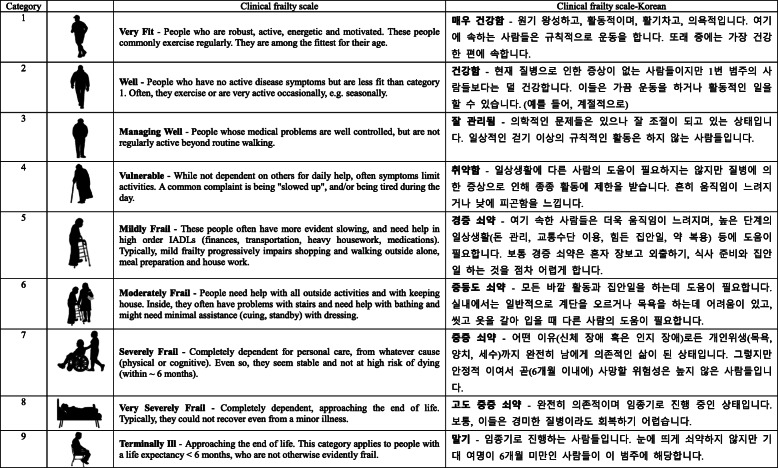
Original and Korean versions of Clinical Frailty Scale

Original CFS in English consists of a scale from 1 (very fit) to 9 (terminally ill), which is scored by clinical judgment; hence, the last group is technically not frail [2] (Fig. 1). To develop the CFS-K, we obtained copyright permission from Dr. Rockwood, who developed the original CFS. Three bilingual experts translated the CFS to Korean independently; then, it was back translated to English by three independent bilingual experts [26]. After this process, a multidisciplinary team of experts, including intensivists, intensive care unit nurses, an expert in geriatric medicine, behavioral scientists, and clinicians, reviewed and confirmed the instruments’ content (Fig. 1). In addition, a pilot test with five patients confirmed the content validity of the scale (data not shown).

### Measurements

To assess the baseline cognitive function, we used the Korean version of the Mini-Mental State Examination (K-MMSE) [27]. Other demographic and clinical information including comorbidity, admitted location, and primary reason for admission were obtained from the participants’ medical records.

To examine the concurrent validity, we used the ECOG PS, K-FRAIL scale, KG-7, and Korean Frailty Index [22–25, 28]. The ECOG PS scale is a measurement tool used to describe a patient’s level of functioning from 0 to 5, with increasing scores indicating increasing levels of deterioration [22]. The K-FRAIL scale is a screening tool for measuring frailty status using a five-item questionnaire, which ranged 1 to 5, with increasing scores indicating increasing frailty [23]. The KG-7 is a screening tool for geriatric assessment using seven items representing each domain of the geriatric assessment scale, which ranged 0 to 7 [24], with decreasing scores indicating increasing deterioration. The Korean Frailty Index is an eight-item questionnaire that measures frailty in older patients, with scores ranging from 0 to 8, and high scores indicating increasing levels of deterioration [25].

### Statistical methods

Data analyses included descriptive statistics (frequencies, means, and standard deviations) and statistical analyses for assessing frailty. In order to measure the CFS-K’s concurrent validity, Pearson’s coefficients for correlation between CFS and the other scales including the KG-7, K-FRAIL, and Korean Frailty Index, and Spearman’s rank-order for correlation between CFS and ECOG PS were computed. In addition, we calculated the sensitivity and specificity of identifying ECOG 3 or more using the area under the curve (AUC). The performance for identifying frailty was compared between CFS-K and the K-FRAIL, KG-7, and Korean Frailty Index, with Bonferroni’s correction to adjust for multiple comparisons. For the analyses, KG-7 was recorded in reverse to achieve the same direction scores. We used the two way sided *p*-values to compare the AUC of CFS-K with AUC of other frailty screening tests. The significance level was set at 0.05. All analyses were performed using STATA version 15 (Stata Corp LP, College Station, TX, USA).

## Results

### Participant characteristics

The participants’ characteristics are demonstrated in Table 1. A total 100 outpatient (*n =* 10, 10.0%) and inpatient (*n =* 90, 90.0%) participants were recruited at two medical centers. The mean age of the study participants was 75.6 years old and 63.0% were men. The mean body mass index was 21.7 kg/m^2^ (standard deviation [SD], 3.5 kg/m^2^). Among the participants, the common comorbidity was chronic lung disease including chronic obstructive pulmonary disease, asthma, and interstitial lung disease (44%), followed by hypertension (38%) and cancer (32%). For the 90 inpatients, the primary reason for admission was pneumonia (42.2%) followed by cancer-related management (24.4%). Of the 10 outpatients, 9 visited for pulmonary disease (90%), and one visited for cardiovascular disease (10%). K-MMSE was measured in 96 (96%) patients and the mean score of the K-MMSE was 22.2 (SD 7.2).

**Table 1:** Characteristics of the study participants

	Participants (***N*** = 100)
**Age (years)**	75.6 (7.0)
**Sex**	
Male	63 (63.0)
Female	37 (37.0)
**Body mass index (kg/m**^**2**^**)**	21.7 (3.5)
**Comorbidity**	
Chronic lung disease	44 (44.0)
Hypertension	38 (38.0)
Cancer (oncology/hematology)	32 (32.0)
Diabetes	30 (30.0)
Cardiac disease (ischemic/vascular)	26 (26.0)
Cerebrovascular disease	17 (17.0)
Chronic kidney disease	11 (11.0)
**Location**	
Outpatient	10 (10.0)
Inpatient	90 (90.0)
**Primary admission cause in ward patients (*N*** **= 90)**	
Pneumonia	38 (42.2)
AE of underlying lung disease	11 (12.2)
Cardiac disease	14 (15.6)
Other infection	5 (5.6)
Cancer related problems	22 (24.4)
**K-MMSE score**^**a**^	22.2 (7.2)

*K-MMSE* Korean version of the Mini-Mental State Examination

Values are mean (SD) or number (%)

^a^Data were obtained from 96 (96%) participants

### Characteristics by clinical frailty scale-Korean

All of the participants completed the CFS-K, and the mean score was 4.8 (SD, 2.5). The characteristics of the participants are grouped by CFS-K scale (Table 2). Except for patients assigned to scale 9 (terminally ill), the mean age and proportion of males were different for each scale but without significance (P for trends 0.576 and 0.052, respectively). Body mass index was higher in patients assigned to scales 1 (very fit)–4 (vulnerable) than in those assigned to scales 5 (mildly frail)–8 (very severely frail), at a significant level (P for trend 0.013). The K-MMSE data were obtained from 96 (96%) of all the participants. The patients assigned to scale 1 (very fit) had the highest (mean (standard deviation [SD])) K-MMSE score (28.7 (2.7)) and those assigned to scale 8 (very severely frail) had the lowest K-MMSE score (12.7(8.1)) with significant difference across the groups (*P* for trends < 0.001). The body mass index of patients assigned to scale 9 (terminally ill) was higher than that of patients assigned to scale 2 (well) and the K-MMSE score (21.0 (8.7)) of patients assigned to scale 6 (moderately frail) (18.9 (5.9)) was higher than that of patients assigned to other scales.

**Table 2:** Characteristics of the study participants as per Clinical Frailty Scale-Korean

	1 Very fit	2 Well	3 Managing well	4 Vulnerable	5 Mildly frail	6 Moderately frail	7 Severely frail	8 Very severely frail	9 Terminally ill
**No. of patients**	10	12	11	16	12	10	10	11	8
**Age, years**	73.2 (6.6)	78.7 (8.3)	74.3 (3.6)	72.8 (6.9)	76.7 (7.0)	77.8 (7.9)	76.9 (8.0)	75.5 (7.7)	75.8 (4.6)
**Male, n**	7 (70.0)	9 (75.0)	5 (45.5)	13 (81.2)	9 (75.0)	5 (50.0)	3 (30.0)	5 (45.5)	7 (87.5)
**Body mass index (kg/m**^**2**^**)**	23.5 (1.1)	21.6 (4.2)	22.8 (2.7)	22.4 (2.9)	20.3 (3.3)	21.2 (3.9)	21.0 (5.3)	19.8 (2.3)	23.0 (3.0)
**K-MMSE**									
Mean (SD)	28.2 (2.7)	23.5 (7.9)	24.5 (4.0)	25.8 (3.5)	24.4 (3.6)	18.9 (5.9)	18.3 (7.3)	12.7 (8.1)	21 (8.7)
Median (IQR)	29 (27–30)	25 (23.5–27.5)	26 (22–28)	27 (25.5–28)	25.5 (22–27)	17 (16–25)	15.5 (13–26)	13 (7–20)	24 (21.5–25)

*K-MMSE* Korean version of the Mini-Mental State Examination

Values in the Table are mean (SD), median (IQR), or number (%)

*P* for trends for body mass index (*p =* 0.013) and K-MMSE (*p <* 0.001) were statistically significant. *p* for trends for age (*p =* 0.576) and sex (*p =* 0.052) were not significant. We excluded participants who were assigned to scale 9 (terminally ill) on the Clinical Frailty Scale-Korean

### Correlation between frailty measures and clinical frailty scale-Korean and validation

The frailty scores by CFS-K are summarized in Table 3. The patients assigned to scale 1 (very fit) had the highest KG7 score (6.7 (0.7)) and the lowest scores on the K-FRAIL (0.3 (0.5)) and Korean Frailty Index (1.2 (1.3)). All of the patients with CFS-K 1 (very fit) showed ECOG PS grade 0 (80%) or 1 (20%). In contrast, the patients assigned to scale 8 (very severely frail) had the lowest KG7 score (0.8 (1.0)) and the highest score of K-FRAIL (3.7 (0.5)) and Korean Frailty Index (6.8 (1.1)). Patients with CFS-K 8 showed ECOG PS grade 3 (30%) or 4 (70%). Regarding patients assigned to scale 9 (terminally ill), the mean (SD) of KG7 (3.9 (2.0)), K-FRAIL (2.9 (1.2)), and Korean frailty index (4.9 (1.5)) were similar to those assigned to scale 4 (vulnerable) or 5 (mildly frail). The ECOG PS scores were inconsistent among patients assigned to scale 9 (terminally ill). The CFS-K scores were positively correlated with K-FRAIL (R = 0.8053) and Korean Frailty Index (R = 0.7742), ECOG PS (R = 0.9190) scores and negatively correlated with KG-7 (R = − 0.8846) scores, except in the case of patients assigned to scale 9 on the CSF-K (terminally ill).

**Table 3:** Performance of K-CFS against that of K-FRAIL, KG-7, Korean frailty index, and ECOG and Pearson’s correlations between K-CFS and other scales

	1 Very fit	2 Well	3 Managing well	4 Vulnerable	5 Mildly frail	6 Moderately frail	7 Severely frail	8 Very severely frail	9 Terminally ill	R^**a**^	R_**s**_^**a**^
**K-FRAIL**										0.8053^*^	0.8048^*^
Mean (SD)	0.3 (0.5)	0.7 (0.8)	0.8 (1.3)	1.3 (0.9)	3.3 (0.9)	3.8 (0.4)	3.5 (1.0)	3.7 (0.5)	2.9 (1.2)		
Median (IQR)	0 (0–1)	0.5 (0–1)	0 (0–1)	1 (1–2)	3.5 (3–4)	4 (4–4)	4 (3–4)	4 (3–4)	3 (2–3.5)		
**KG-7**										−0.8846^*^	−0.8860^*^
Mean (SD)	6.7 (0.7)	5.4 (1.2)	5.5 (1.0)	4.3 (1.1)	3.4 (0.7)	1.8 (1.0)	1.0 (1.2)	0.8 (1.0)	3.9 (2.0)		
Median (IQR)	7 (7–7)	5 (5–6.5)	6 (5–6)	4 (3–5)	3.5 (3–4)	1.5 (1–2)	1 (0–1)	1 (0–1)	3.5 (2–5.5)		
**Korean frailty index**										0.7742^*^	0.7883^*^
Mean (SD)	1.2 (1.3)	3.3 (1.4)	2.2 (1.4)	4.3 (1.4)	5.8 (1.1)	5.2 (0.9)	6.2 (1.2)	6.8 (1.1)	4.9 (1.5)		
Median (IQR)	1 (0–2)	3 (2–5)	3 (1–3)	4 (3–5.5)	6 (5.5–6)	5.5 (4–6)	6.5 (5–7)	7 (6–8)	5 (3.5–6)		
**ECOG PS**										0.9190^*^	0.9184^*^
0	8 (80.0)	3 (25.0)	2 (18.2)	1 (6.3)	0	0	0	0	1 (12.5)		
1	2 (20.0)	9 (75.0)	9 (81.8)	13 (81.3)	1 (8.3)	1 (8.3)	0	0	2 (25.0)		
2	0	0	0	2 (12.5)	8 (66.7)	8 (66.8)	2 (20.0)	0	4 (50.0)		
3	0	0	0	0	3 (25.0)	8 (80.0)	8 (80.0)	3 (30.0)	1 (12.5)		
4	0	0	0	0	0	0	0	7 (70.0)	0		

*K-CFS* Clinical Frailty Scale-Korean, *K-FRAIL* Korean version of the fatigue, resistance, ambulation, illness, and loss of weight, *KG-7* Korean Cancer Study Group Geriatric Score, *ECOG PS* Eastern Cooperative Oncology Group Performance Status

R and R_s_ were calculated using Pearson correlation and Spearman correlation, respectively

*P* for trends for all the variables were statistically significant (*p <* 0.001)

We excluded participants who were assigned to scale 9 (terminally ill) on the Clinical Frailty Scale-Korean

^*^p < 0.01

**Fig. 2:**
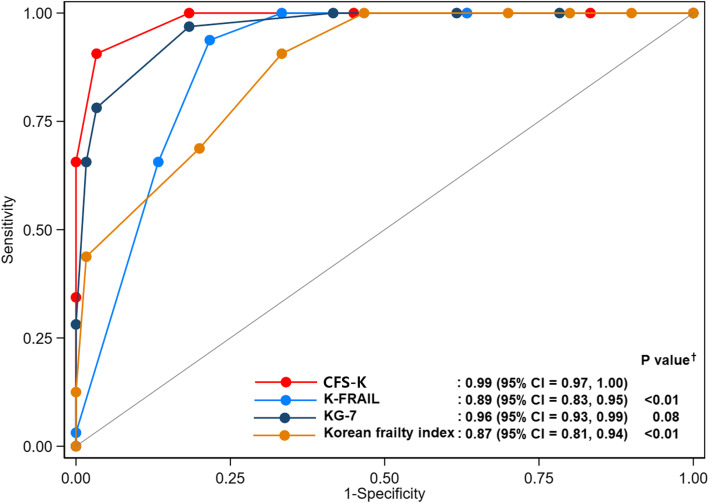
Receiver operating characteristic (ROC) curves of frailty measures for identifying ECOG PS 3 or more (*N* = 92). In this figure, KG-7 was recorded as reverse to achieve the same direction scores. The participants assigned to CFS-K scale 9 (terminally ill) were excluded in this analysis. ^†^*P* values were obtained to compare the ROC curves, and Bonferroni’s correction was used to adjust for multiple comparisons

Regarding the receiver operating characteristic (ROC) curve for identifying ECOG PS grade 3 or more, the CFS-K showed better performance (AUC = 0.99) than the KG-7 (AUC = 0.96; *p =* 0.08), K-FRAIL (AUC = 0.89; *p <* 0.01), and Korean Frailty Index (AUC 0.87; *p <* 0.01) (Fig. 2). In addition, the CFS-K has a sensitivity of 90.6% and a specificity of 97.0% for identifying ECOG PS grade 3 or more.

## Discussion

In this study, we translated the CFS into Korean and evaluated the content and concurrent validity by comparing it with other scales, namely the ECOG PS, K-FRAIL, KG-7, and Korean Frailty Index. The patients were subjected all of the scales regardless of age or sex. High scores on the CFS-K were correlated to low body mass index and low K-MMSE score. The CFS-K scores were significantly correlated with the scores of other scales and showed the best assessment of frailty.

The CFS-K was found to be a useful screening tool of frailty in Korean older patients. In this study, the newly translated Korean version of CFS recognized frail patients more effectively than other scales. Previous studies have suggested the CFS is a useful screening tool based on clinical judgement for measuring frailty [8, 14]. the translated version was also administered successfully. Moreover, the CFS-K correlated well with other scales including the ECOG PS, K-FRAIL, KG-7, and Korean Frailty Index, which are already used in clinical settings. Previous study showed that CFS has a sensitivity of 56.0% and a specificity 98.4% for identifying frail according to the definition of CHS frailty screening. In this study, the CFS-K also investigated concurrent validity. With the AUC of 0.99, a sensitivity 90.6%, and a specificity of 97.0%, the CFS-K showed excellent performance for identifying ECOG PS grade 3 or more. As the CFS has predictive and prognostic features with regard to clinical outcomes in various clinical settings [5, 16, 17], the CFS-K could be a useful screening tool for frail older in South Korea and also help provide optimal management.

Interestingly, the body mass index and K-MMSE score showed significant differences across the CSF-K scales. The relationship between frailty and sarcopenia has been reported in several studies [29, 30]; this study showed consistent results. Since low body mass index is an important risk factor of poor prognosis [31, 32], high scores on the CFS-K would be associated with poor clinical outcomes. The association between frailty and cognitive decline has been reported, and the results were consistent with previous findings [33, 34]. As important clinical characteristics can be distinguished by quick assessment with the CFS-K, it can be a valuable tool for frailty screening.

The patients assigned to scale 9 (terminally ill) showed unique characteristics. Because of the definition, for patients who were not evidently frail but had less than 6 months’ life expectancy, the scores of frailty indexes were between scale 4 (vulnerable) and scale 5 (mildly frail) and the ECOG PS score also ranged from 0 to 3; their body mass index and K-MMSE scores were also relatively high. In this study, the patients diagnosed with advanced solid or hematologic malignancy with high tumor burden were assigned to scale 9 (terminally ill). Nowadays, the life expectancy is increasing due to improvement in cancer treatment, organ transplantation, and critical care with organ-supporting systems [35–39]. Further studies regarding scale 9 (terminally ill) patients’ clinical outcomes and prognosis are warranted.

This study has some limitations. First, the validation of the CFS-K was performed with a relatively small number of participants. Second, this study included outpatients and inpatients, not a community-based population. Moreover, only patients who gave consent were enrolled. These factors might have caused selection bias. Further large-scale studies with general population and patients in various clinical settings are warranted.

## Conclusions

In conclusion, the CFS-K is a valid scale for measuring frailty in older Korean patients. The CFS-K scores were significantly correlated with the scores of other scales. To evaluate the predictive and prognostic value of this scale, further larger-scale studies in various clinical settings are warranted.

## Data Availability

The data that support the findings of this study are available on request from the corresponding author. The data are not publicly available due to privacy or ethical restrictions.

## Abbreviations

FRAIL: Fatigue, Resistance, Ambulation, Illness, and Loss of weight
CHS: Cardiovascular Health Study
CFS: Clinical Frailty Scale
ECOG PS: Eastern Cooperative Oncology Group Performance Status
K-FRAIL: Korean version of the fatigue, resistance, ambulation, illness, and loss of weight
KG-7: Korean Cancer Study Group Geriatric Score
CFS-K: Korean version of Clinical Frailty Scale
K-MMSE: Korean version of the Mini-Mental State Examination
AUC: Area under the curve
SD: Standard deviation
ROC: Receiver operating characteristic
